# Both Short and Long Sleep Durations Are Associated with Poor Cognition and Memory in Chinese Adults Aged 55+ Years—Results from China Health and Nutrition Survey

**DOI:** 10.3390/life12111798

**Published:** 2022-11-06

**Authors:** Yingting Cao, Xiaoyue Xu, Ming Li, Jianghong Liu, Zumin Shi

**Affiliations:** 1 Non-Communicable Diseases and Implementation Science Laboratory, Baker Heart and Diabetes Institute, Melbourne, VIC 3004, Australia; 2 Melbourne School of Population and Global Health, The University of Melbourne, Melbourne, VIC 3010, Australia; 3 School of Population Health, University of New South Wales, Sydney, NSW 2052, Australia; 4 Centre for Population Health Research, Division of Health Sciences, University of South Australia, Adelaide, SA 5005, Australia; 5 Family and Community Health, University of Pennsylvania School of Nursing, Philadelphia, PA 19104, USA; 6 Human Nutrition Department, College of Health Sciences, QU Health, Qatar University, Doha 2713, Qatar

**Keywords:** cognition, memory and memory decline, sleep duration, older population, socioeconomic factors

## Abstract

**Simple Summary:**

Sleep plays an important role in cognition and memory in older adults and elderly populations. Rapid ageing in low-and middle-income countries (LMICs), including China, comes with the great challenge of the increased prevalence of cognition decline and dementia, consequently leading to an increase in chronic disease burdens. However, most studies that investigate the association between sleep and cognition are carried out in high-income countries and are limited to cross-sectional studies, whereas 80% of the world’s older population is estimated to live in LMICs in 2050. To fill in the gap, we aim to examine the associations between sleep duration and cognitive functions and memory in older Chinese adults in 2004, 2006, and 2015, using the China Health and Nutrition Survey. We found a U-shaped relationship between sleep duration, cognition, and memory, with a short and long sleep duration being associated with a 23% and 47% increase in cognitive decline and 63% and 48% increase in poor memory, respectively. These associations were not modified by demographic features such as education, income, and urbanity. These findings confirmed the importance of maintaining a normal sleep duration for cognition function. Our results may implicate the need for potential prevention strategies in reducing cognition decline and dementia in other LMIC populations.

**Abstract:**

We aimed to examine the associations between sleep duration and cognitive functions and memory in older Chinese adults attending the China Health and Nutrition Survey. A total of 7924 participants 55 years and older who reported their sleep duration and had a cognitive screen test in 2004, 2006, and 2015 were included in the analysis. Mixed-effects logistic regression models were used to assess the associations. A short sleep duration (≤6 h/day) and long sleep duration (≥10 h/day) were positively associated with a low global cognitive score (odds ratio—OR: 1.23, 95% CI: 1.01–1.50; OR: 1.47, 95% CI: 1.17–1.79, respectively). Both short sleepers and long sleepers had an increased risk of self-reported poor memory (OR: 1.63, 95% CI: 1.39–1.91; OR: 1.48, 95% CI: 1.25–1.74, respectively). No differences in the above associations were found for income, education, and urbanity. In conclusion, both the short and long sleep duration were associated with declined cognition and memory. Maintaining a normal sleep duration may aid in the prevention of cognitive function decline in older adults.

## 1. Introduction

The ageing population has been continually increasing globally. The World Health Organization predicts that by 2030, 1 in 6 people worldwide are projected to be 60 years or older [1]. Ageing has become a global burden, as it remains the strongest risk factor for poor memory, cognition impairment, and neurodegenerative dementias [2]. In China, a meta-analysis showed that the pooled prevalence of mild cognitive impairment among the population aged 55 and over was 15.4% [3]. Strategies to target key risk factors (e.g., lifestyle, clinical, and social) to prevent cognitive impairment and further delay dementia onset are suggested to be prioritized for public health [4]. 

Among the various risk factors, sleep plays an important role in cognitive performance [5]. A systematic review and meta-analysis suggested that both a short and long sleep duration are associated with poor cognitive performance [6]. Adverse changes in sleep duration over several years are associated with poorer cognitive function [7]. However, those studies were mostly conducted in high-income countries. Whether these associations exist in low- and middle-income countries (LMICs), where 80% of world’s older population will reside in 2050 [1], is yet to be determined. It is critical to understand the association between sleep duration and cognition in China particularly, as the country is undergoing a rapid economic and epidemiological transition [8] that includes a fast-ageing population and, consequently, increasing disease burdens [9]. Lifestyle factors, such as smoking [10] and alcohol consumption [11], are associated with both sleep patterns and cognitive function. Understanding the implications of sleep on cognitive function will aid other LMICs in their epidemiological transitions to tackle ageing-related problems.

In China, the prevalence of short (<7 h/day) and long (>8 h/day) sleep durations among older adults was 42.3% and 17.6%, respectively [12]. However, only a few studies have assessed the association between sleep duration and cognitive function among older adults. In the study from northern China (Hebei), a U-shaped association was found between sleep duration and cognitive impairment among adults aged 40 years and older [13]. The study from southern China (Guangdong), which was completed with a 4.1-year follow-up, concluded that sleeping less than 5 h/day was associated with a 50% increased risk of developing memory impairment among those aged 50 years and older [14]. However, it is unknown whether the findings on the association between sleep duration and cognitive function also exist in other regions of China. 

An increasing number of studies have shown that climate and lifestyle are associated with sleep pattern, particularly in the elderly [15]. China is a very diverse nation, with large differences in geography, climate, and lifestyle in the north, south, central, and coastal areas, which may affect sleep pattern and quality. For example, because the weather in the north is much colder in winter, people may go to bed earlier, whereas people from the south tend to go to bed and wake up later. Some parts of China eat chili peppers as one of the main dishes, whereas other parts of the country drink a lot of tea [16,17], all of which have been previously demonstrated by our team to be related to cognition in the elderly. 

Therefore, this study aims to assess the association between sleep duration and cognitive functions in adults aged 55 years and above who attended the China Health and Nutrition Survey (CHNS), which covers 15 provinces and municipal cities in China. This study utilized repeated measures of sleep duration and cognitive function and took into consideration the possible disparities in socioeconomic factors, as well as a variety of other potential confounders.

## 2. Materials and Methods

### 2.1. Study Design and Study Sample

This is an association study based on repeated measurements of sleep time and cognition function among adults aged 55+ years from the CHNS. The CHNS is an ongoing, open, prospective, household-based cohort study conducted in 15 provinces and municipal cities in China [18]. The CHNS uses a multistage, random-cluster sampling process to select samples in both urban and rural areas. Ten waves of data collection (i.e., 1989, 1991, 1993, 1997, 2000, 2004, 2006, 2009, 2011, and 2015) were conducted. 

In the 2004, 2006, and 2015 surveys, cognitive screen tests were conducted among those aged ≥55 years. In 2015, participants from three megacities (Beijing, Shanghai, and Chongqing) joined the survey for the first time. In total, 7924 participants attended the cognitive screen tests and reported their sleep duration. Of these participants, 2889 attended the screen test in at least two surveys. Participants who completed at least one cognitive screen test were included in the analysis. Sleep duration surveys were also measured in the same time frames. 

The survey was approved by the institutional review committees of the University of North Carolina and the National Institute of Nutrition and Food Safety (China). Informed consent was obtained from all participants. The response rate based on those who participated in the 1989 and 2006 survey was >60%. 

### 2.2. Outcome Variable: Cognitive Function and Memory

The cognitive screening items used in the CHNS included a subset of items from the Telephone Interview for Cognitive Status-Modified [19]. The cognition assessment tool has been used in other population studies in China to assess cognitive function [20]. The cognitive screening included an immediate and delayed recall of a 10-word list (score of 10 for both recall attempts), counting backward from 20 (score of 2), and serial 7 subtraction (score of 5). The total global cognitive score ranges from 0 to 27. A high cognitive score represents better cognition. The cognitive function test started with the immediate recall of a 10-word list. The interviewer (i.e., trained health worker) read ten words at a speed of two seconds per word. The participants were given two minutes to memorize the ten words. For each correct recalled word, a score of 1 was given. The participants were then asked to count back from 20 to 1. If the participants made a mistake in the first try, a second chance was given. A score of 2 was given to those who answered correctly on the first try, or 1 for the second try. After the count test, the participants were asked to do five consecutive subtractions of 7 from 100. Each correct subtraction was given a score of 1. Finally, the participants were asked to recall the 10-word list that was tested beforehand. Each recalled word was given a score of 1. In the current study, we chose the first quintile of the cognitive function test score as representing poor cognitive function, which corresponds to a global cognitive function score cut-off of <7 as used in other studies [21].

The self-reported memory status was assessed by the questions: “How is your memory? (1) very good; (2) good; (3) OK; (4) bad; (5) very bad; (6) unknown” and “In the past twelve months, how has your memory changed? (1) improved; (2) stayed the same; (3) deteriorated; (4) unknown”. Participants were recorded as having poor memory if they answered “bad, or very bad” to the question. Memory decline was defined if the answer to the question was “deteriorated”.

### 2.3. Exposure Variable: Sleep Duration

Sleep duration was assessed from surveys in 2004, 2006, and 2015 with a self-reported questionnaire asking: “How much time each day do you usually spend in bed either asleep or lying there including nighttime? (hours).” Responses to the number of hours per day ranged from 1 to 18 h. In the analysis, we categorized sleep duration as short (≤6 h), normal (7–9 h), and long (≥10 h). In addition to the use of categorical short and long sleep duration, we also used sleep duration as a continuous variable to assess the nonlinear association between sleep and cognition.

### 2.4. Covariates 

Potential covariates considered include socioeconomic status, lifestyle factors, and physical health, with the first two categories being collected in each wave by using a structured questionnaire.

Socioeconomic status: Age, sex, education (low: illiterate/primary school; medium: junior high school; and high: senior high school or higher), and urbanization levels [8] (recoded into tertiles as low, medium, and high).

Physical health: Height, weight, and blood pressure were measured at each wave. Overweight or obesity was defined as a body mass index (BMI, kg/m^2^) ≥ 24. Hypertension was defined as systolic blood pressure ≥ 140 mm Hg, diastolic blood pressure ≥ 90 mm Hg, or having known hypertension. Self-reported diabetes and stroke were coded as yes or no.

Lifestyle factors: Physical activity, smoking, and alcohol drinking. Specifically, the physical activity level (metabolic equivalent of task—MET) was estimated on the basis of self-reported activities (including occupational, domestic, transportation, and leisure-time physical activity) and duration using the Compendium of Physical Activities [22]. Smoking status was categorized as non-smokers, ex-smokers, and current smokers. Alcohol drinking was categorized as either yes or no. 

### 2.5. Statistical Analysis

The chi-square test was used to compare differences between groups by sleep duration for categorical variables and an analysis of variance (ANOVA) was used for continuous variables. A mixed-effects logistic regression model using the melogit command in Stata (StataCorp) was applied to assess the association between sleep duration and poor cognitive function, poor memory, or memory decline. The poor cognition was modelled as a function of fixed effects of sleep, whereas individual participant variables were modelled as random effects. Both the intercept and slope were fitted with random-effects components to account for interindividual differences in baseline measures and the rate of change [23]. A set of models was used: model 1 adjusted for age and gender; model 2 further adjusted for smoking, alcohol drinking, urbanization, and education; model 3 further adjusted for physical activity; and model 4 further adjusted for BMI and hypertension. In the analyses, a sleep duration of 7–9 h was used as the reference group.

To assess the nonlinear association between sleep and cognitive function, we fitted mixed-effects models by putting the sleep duration and the square of the sleep duration in the multivariable models. The margins command in Stata was used to estimate the marginal probability (95% CI) of poor cognition, self-reported poor memory, and self-reported memory decline and the mean (95% CI) score of cognitive function, which represent the adjusted values based on the multivariable models. Subgroup analyses were performed to examine whether the association between sleep duration, cognition, and memory differs by socioeconomic factors (e.g., income, education, and urbanity) by adding a multiplicative term in the model. The results were visually presented using the margins plot command. All the analyses were performed using STATA 17.0 (StataCorp). Significance was considered when *p* < 0.05 (two-sided).

## 3. Results

In total, 7924 participants who provided sleep and cognition data in 2004, 2006, and 2015 were analysed. Of the participants, 2889 had information on sleep and cognition from at least two survey years. The mean sleep duration was 8.20 h at baseline (2004), 8.08 h at in 2006, and 7.1 h in 2015. The prevalence of short and long sleep duration was 12.9% and 20.6%, respectively, in 2004. The mean total cognitive function scores were 13.0 (SD 6.5) in 2004, 12.4 (SD 6.6) in 2006, and 13.8 (SD 6.7) in 2015. Baseline subject characteristics are shown in Table 1**.** In brief, age was associated with abnormal sleep duration. Compared with those who had normal sleep, those with a short sleep or long sleep duration were older. Similarly, education and urbanization levels were also associated with sleep duration. Participants who had a lower education and lived in low-urbanization areas were more likely to have abnormal sleep durations than their counterparts.

Table 2 shows the association between sleep duration, cognition, and memory. In the final adjusted model (model 4), compared with normal sleep duration, the odds ratio (OR, 95% CI) for poor cognition was 1.23 (95% CI: 1.01–1.50) for a short sleep duration and 1.47 (95% CI: 1.17–1.79) for a long sleep duration. Both short sleepers and long sleepers had an increased OR for self-reported poor memory (OR: 1.63 (95% CI: 1.39–1.91)) in short sleepers and an OR of 1.48 (95% CI: 1.25–1.74) in long sleepers. 

**Figure 1:**
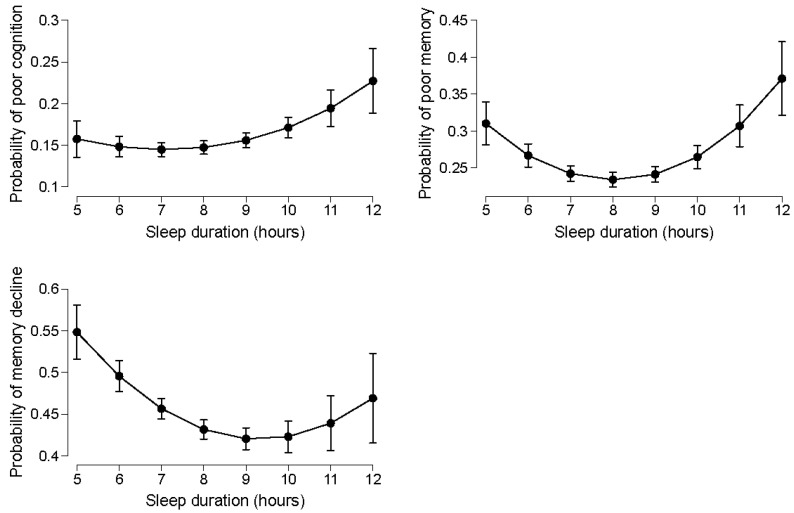
Nonlinear association between sleep duration and poor cognition, self-reported poor memory, and self-reported memory decline. Values are means (95% CI) from the marginal mean probability found through the mixed logistic model that are adjusted for age, gender, education, income, urbanization, smoking, alcohol drinking, and physical activity.

The nonlinear association between sleep duration and poor cognition, self-reported poor memory, and self-reported memory decline is presented in Figure 1, after adjusting for age, gender, socioeconomic, and health-behaviour factors. There was a U-shaped association between sleep duration and cognitive function, including self-reported poor memory, self-reported memory decline, and poor cognition, based on the global cognition score.

**Figure 2:**
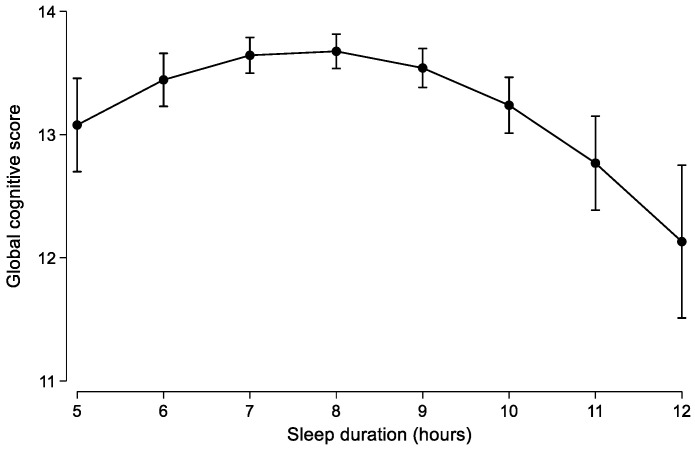
Nonlinear association between sleep duration and global cognitive score. Values are means (95% CI) from the linear mixed model adjusted for age, gender, education, income, urbanization, smoking, alcohol drinking, and physical activity.

There was also a U-shaped, nonlinear association between sleep duration and global cognitive scores (Figure 2). There was no difference between sleep duration, cognition, and memory for different levels of income, education, and urbanity in the current study sample (data not shown).

## 4. Discussion

The present study examined the relationship between sleep duration and cognition in 7924 older adults using a representative CHNS cohort of the older Chinese population. The study indicated a U-shaped relationship between sleep duration, cognition, and memory, in which both short (≤6 h/day) and long sleep durations (≥10 h) were associated with a declined global cognitive score (<7). Self-reported memory and memory decline were worse among subjects with both short and long sleep durations compared to those with normal sleep durations (7–9 h/day). The associations between sleep duration, cognition, and memory were not different among the varying levels of income, education, and urbanity. 

Similar U-shaped associations between sleep duration and cognition have been observed in western countries [24,25] and some regions of China [14,26]. Our study confirmed the U-shaped association between sleep duration and cognitive function in these studies by using well-established cohorts and taking into consideration the socioeconomic disparities across different regions of China. The U-shaped association between sleep duration, cognition, and memory did not differ for varying levels of income, education, and urbanity in the sampled spectrum of varying demographics and socioeconomic conditions that were covered in this study. Evidence suggests that ethnicity and socioeconomic status could modify the associations between sleep and cognition in U.S. African children, which may be primarily due to family adversity and discrimination [27]. Yet, little is evident on whether socioeconomic factors would modify the association between sleep and cognition in the older population. Research has increasingly focused on sleep health disparities among adults that results in cognitive decline and how racism, the environment, and other sociocultural factors may causally relate to these sleep health disparities [28]. However, we did not find interactions between sleep duration and socioeconomic factors in relation to cognition and memory in our study with the Chinese population. It is likely that sleep habits have already been established through adulthood, and the association with cognition and memory may not likely be affected by levels of socioeconomic factors. Nevertheless, future research needs to explore other environmental factors, such as neighbourhood and community upbringing, which has been shown to contribute to sleep and cognition across one’s lifespan.

The exact mechanism underlying the U-shaped link between sleep duration and cognitive function in older adults is not fully understood. Several possible pathways have been suggested. A short sleep duration may have negative effects on brain morphometry, activation, and physiology [29,30]. A short sleep duration has also been found to be associated with the accumulation of beta-amyloid in the brain [31,32], which is a hallmark of Alzheimer’s disease [33,34]. A long sleep duration (>9 h), on the other hand, has been shown to cause a higher risk for incident all-cause dementia over a mean period of 13 years leading up to baseline, indicating an early biological marker of neurodegeneration [35]. In addition, extreme sleep durations may be associated with circadian misalignment and disruption, which has been evident in patients with mild cognition impairment [36,37]. The internal circadian system has been suggested to regulate cognition by interacting with the sleep–wakefulness regulatory process, and any changes in sleep, circadian processes, and circadian misalignment can have important implications for cognitive function [38,39]. Disruption of the circadian rhythm can lead to circadian syndrome which includes metabolic abnormalities (e.g., elevated blood glucose, obesity, hypertension, dyslipidemia) and depression [40]. All these conditions have been found to be associated with cognitive function. 

There are several strengths and limitations that need to be acknowledged. One of our study’s strengths is that we examined the association between sleep duration and the potential risk of cognitive decline and dementia using a representative Chinese cohort while considering the disparities in socioeconomic factors across different regions in China. We also controlled for a variety of potential confounding variables including lifestyle, BMI, and several major chronic disorders in addition to educational level and region. Nevertheless, limitations of this study should be considered. Firstly, sleep duration was based on the self-reported total time in bed, including sleeping or lying there. It is a proxy for sleep duration, as most likely, the time reported was for sleep. It has been found that self-reported sleep does not correspond closely with objective measures of sleep when assessed using actigraphy [41]. However, self-reported measures seem to be practical and cost-effective for epidemiological studies, particularly for a large sample size. Moreover, poor memory and memory decline were also self-reported; thus, it is possible that those who had normal ageing-related memory decline reported themselves as having “poor memory,” leading to an overestimation. Thirdly, we do not have information on daytime napping, which may be associated with cognition and memory. However, the association between napping and cognition is inconclusive as some studies indicate a decrease in cognitive function, whereas a more recent study suggests an increase in cognitive function [42,43].

## 5. Conclusions

In this study, we found that both short and long sleep durations were associated with poor cognition and memory across different regions in China among the older population, with no difference in levels of income, education, and urbanity. Several biological and physiological explanations for the association between extreme sleep duration and cognition in older adults have been explored. Given that both poor sleep health and declining cognitive functioning in older adults have been increasing in prevalence, and are likely to result in increased mortality and morbidity, our results may shed light on the need for prevention strategies. More specifically, around 30% of all participants in our study reported a short or long sleep duration, which contributes to cognitive decline and demonstrates the need for sleep-related lifestyle changes with the ultimate goal of reducing the onset of cognitive decline in older populations. Our findings may guide health professionals to consider using sleep assessments and sleep education for routine patient care. This can include encouraging a healthy sleep routine, avoiding extreme sleep durations, and implementing universal interventions that would not be affected by geographical regions and socioeconomic factors. A normal sleep duration is vital for maintaining good health in individuals and further research needs to be conducted on factors that may contribute to poor sleep and the resulting cognitive decline, particularly in LMIC settings.

## Figures and Tables

**Table 1:** Sample characteristics by sleep duration among adults who attended the first cognition test in the China Health and Nutrition Survey (N = 7924).

	≤6 h	7–9 h	≥10 h	*p*-Value *
	N = 1184	N = 5669	N = 1071	
Age (years)	64.9 (7.7)	63.1 (7.2)	66.9 (8.9)	<0.001
Sex				0.026
Men	533 (45.0%)	2747 (48.5%)	484 (45.2%)	
Women	651 (55.0%)	2922 (51.5%)	587 (54.8%)	
Education				<0.001
Low	620 (52.5%)	2867 (50.7%)	768 (71.9%)	
Medium	302 (25.5%)	1408 (24.9%)	173 (16.2%)	
High	260 (22.0%)	1383 (24.4%)	127 (11.9%)	
Urbanization				<0.001
Low	139 (11.8%)	912 (16.2%)	301 (28.1%)	
Medium	263 (22.3%)	1296 (23.0%)	310 (29.0%)	
High	779 (66.0%)	3435 (60.9%)	459 (42.9%)	
Smoking				0.91
Non-smoker	822 (69.5%)	3940 (69.6%)	753 (70.5%)	
Ex-smoker	65 (5.5%)	301 (5.3%)	61 (5.7%)	
Current smoker	295 (25.0%)	1419 (25.1%)	254 (23.8%)	
Alcohol drinking	306 (26.1%)	1556 (27.6%)	270 (25.3%)	0.21
Survey year				<0.001
2004	364 (30.7%)	1886 (33.3%)	593 (55.4%)	
2006	126 (10.6%)	696 (12.3%)	138 (12.9%)	
2015	694 (58.6%)	3087 (54.5%)	340 (31.7%)	
Physical activity (MET)	63.8 (82.3)	75.0 (87.8)	63.5 (84.8)	<0.001
BMI (kg/m^2^)	24.1 (3.6)	24.0 (3.5)	23.3 (3.9)	<0.001
BMI ≥ 24 (kg/m^2^)	524 (47.5%)	2529 (47.6%)	396 (40.2%)	<0.001
Hypertension	543 (47.9%)	2259 (41.7%)	499 (48.8%)	<0.001
Diabetes	94 (8.0%)	329 (5.8%)	64 (6.0%)	0.021
Stroke	27 (2.3%)	131 (2.3%)	46 (4.3%)	<0.001
Poor memory	344 (29.3%)	1097 (19.5%)	382 (35.9%)	<0.001
Memory decline	595 (52.0%)	2218 (40.1%)	519 (50.0%)	<0.001
Global cognitive score < 7	190 (16.0%)	732 (12.9%)	300 (28.0%)	<0.001

* One-way ANOVA was used for variables: age, physical activity, and BMI; chi-square test was used for other categorical variables listed in the table.

**Table 2:** Odds ratio (95% CI) for global cognitive score below 7, self-reported poor memory, and self-reported memory decline based on sleep duration among Chinese adults aged >= 55 years old; China Health and Nutrition Survey (N = 7924).

	≤6 h	7–9 h	≥10 h
**Global cognitive score < 7**			
Model 1	1.13 (0.96–1.34)	1.00	2.06 (1.76–2.41)
Model 2	1.17 (0.99–1.38)	1.00	1.65 (1.41–1.93)
Model 3	1.20 (1.00–1.45)	1.00	1.52 (1.27–1.82)
Model 4	1.23 (1.01–1.50)	1.00	1.47 (1.22–1.79)
Model 4 + ≥2 waves	1.20 (0.93–1.57)	1.00	1.47 (1.17–1.85)
**Self-reported poor memory**			
Model 1	1.57 (1.37–1.80)	1.00	1.96 (1.71–2.25)
Model 2	1.63 (1.42–1.87)	1.00	1.65 (1.44–1.90)
Model 3	1.64 (1.40–1.92)	1.00	1.50 (1.28–1.76)
Model 4	1.63 (1.39–1.91)	1.00	1.48 (1.25–1.74)
Model 4 + ≥2 waves	1.42 (1.15–1.75)	1.00	1.43 (1.18–1.74)
**Self-reported memory decline**			
Model 1	1.51 (1.33–1.70)	1.00	1.31 (1.15–1.49)
Model 2	1.56 (1.38–1.76)	1.00	1.16 (1.02–1.31)
Model 3	1.60 (1.39–1.82)	1.00	1.09 (0.95–1.26)
Model 4	1.63 (1.42–1.88)	1.00	1.10 (0.95–1.28)
Model 4 + ≥2 waves	1.44 (1.19–1.75)	1.00	1.02 (0.86–1.22)

Model 1 adjusted for age and gender. Model 2 further adjusted for smoking, alcohol drinking, income, urbanicity, and education. Model 3 further adjusted for physical activity. Model 4 further adjusted for BMI and hypertension. Model 4 + ≥2 waves represents Model 4 for those who attended at least two waves of the survey.

## Data Availability

The data presented in this study are openly available at https://www.cpc.unc.edu/projects/china (accessed on 5 January 2022).
